# Essential Oil-Rich Chinese Formula Luofushan-Baicao Oil Inhibits the Infection of Influenza A Virus through the Regulation of NF-*κ*B P65 and IRF3 Activation

**DOI:** 10.1155/2021/5547424

**Published:** 2021-08-30

**Authors:** Xin Mao, Shuyin Gu, Huiting Sang, Yilu Ye, Jingyan Li, Yunxia Teng, Feiyu Zhang, Qinhai Ma, Ping Jiang, Zifeng Yang, Weizhong Huang, Shuwen Liu

**Affiliations:** ^1^Guangdong Provincial Key Laboratory of New Drug Screening, School of Pharmaceutical Sciences, Southern Medical University, Guangzhou 510515, China; ^2^Guangdong Provincial Key Laboratory of Gynecological Chinese Medicine, Huizhou 516113, China; ^3^State Key Laboratory of Respiratory Disease, National Clinical Research Center for Respiratory Disease, Guangzhou Institute of Respiratory Health, The First Affiliated Hospital of Guangzhou Medical University, Guangzhou 510230, China

## Abstract

**Background:**

Luofushan-Baicao Oil (LBO) is an essential oil-rich traditional Chinese medicine (TCM) formula that is commonly used to treat cold, cough, headache, sore throat, swelling, and pain. However, the anti-influenza activities of LBO and the underlying mechanism remain to be investigated.

**Methods:**

The *in vitro* anti-influenza activity of LBO was tested with methyl thiazolyl tetrazolium (MTT) and plaque assays. The effects of LBO on the expressions of viral nucleoprotein and cytokines were evaluated. In the polyinosinic-polycytidylic acid- (Poly I: C-) induced inflammation model, the influences of LBO on the expression of cytokines and the activation of NF-*κ*B P65 (P65) and interferon regulatory factor 3 (IRF3) were tested. After influenza A virus (IVA) infection, mice were administered with LBO for 5 days. The lung index, histopathologic change, the expression of viral protein, P65, and IRF3 in the lung tissue were measured. The levels of proinflammatory cytokines in serum were examined.

**Results:**

*In vitro*, LBO could significantly inhibit the infection of IVA, decrease the formation of plaques, and reduce the expression of viral nucleoprotein and cytokines. LBO could also effectively downregulate the expression of interleukin-1*β* (IL-1*β*), interleukin-6 (IL-6), and interferon-*β* and the activation of P65 and IRF3 in Poly I:C-treated cells. In the IVA-infected mice model, inhalation of LBO with atomizer could decrease the lung index, alleviate the pathological injury in the lung tissue, and reduce the serum levels of IL-1*β* and IL-6. LBO could significantly downregulate the expression of viral protein (nucleoprotein, PB2, and matrix 2 ion channel) and the phosphorylation of P65 and IRF3 in the lungs of mice.

**Conclusion:**

The therapeutic effects of LBO on treating influenza might result from the regulation of the immune response of IVA infection. LBO can be developed as an alternative therapeutic agent for influenza prevention.

## 1. Introduction

The influenza virus is one of the most common respiratory pathogens, which is a substantial threat to the world [1]. Influenza virus infections could affect the upper and lower respiratory tract, inducing cough, fever, sore throat, rhinorrhea, and pneumonia [2]. Influenza virus can induce seasonal epidemics and worldwide pandemics. There have been three influenza pandemics in the past hundred years (1957, 1968, and 2009). First reported in Mexico and the United States, the 2009 H1N1 pandemic caused significant morbidity and mortality; about half a million people were dead from it [3, 4]. Children, old people, pregnant women, and people with chronic illness are considered to be high-risk groups that can be easily infected by the influenza virus [5]. With the outbreak of the worldwide pandemic induced by coronavirus (SARS-CoV-2) at the end of 2019, evidence shows that the influenza A virus (IVA) could aggravate SARS-CoV-2 infection. Hence, the prevention of influenza infection is of great significance [6]. Neuraminidase inhibitor, oseltamivir, is the most commonly used antiviral drug in treating influenza. However, with the widespread oseltamivir-resistance gene, the limitation of oseltamivir is getting more serious [7, 8].

Influenza virus can be recognized by many pattern recognition receptors (PRRs), such as retinoic acid-inducible gene I (RIG-I) and Toll-like receptors (TLRs). Expressed in airway epithelial cells, TLR3 is able to detect the dsRNA intermediate by influenza virus [9, 10]. The activation of PRRs initiates innate immune responses, resulting in the releasing of interferons (IFNs), interleukins (ILs), and chemokines, leading to clearance of viruses and infected cells. However, the excessive activation of innate immune also results in lung injury [11]. In recent years, the attenuating of proinflammatory responses and limiting influenza-induced tissue damage has been considered as an alternative strategy for treating influenza virus infection [12, 13].

Luofushan-Baicao Oil (LBO) is a unique essential oil-rich traditional Chinese herbal (TCM) formula composed of seventy-nine kinds of herbs and related extractions: methyl salicylate, peppermint oil, camphor oil, turpentine oil, eucalyptus oil, camphor, menthol, cinnamon oil, *Ocimum gratissimum* oil, borneol, star anise oil, and Baicaojing extraction (tea seed oil extraction of the rest of sixty-eight herbs). LBO originated from Ge-Hong, a famous pharmacist in Jin Dynasty. In the Taoism prescription in Ming Dynasty, the formula of LBO was described. The preparation skill of LBO had been listed in the Chinese intangible cultural heritage in recent years.

According to the theory of TCM, influenza and other epidemics belong to the category of “plague (Wenyi).” The “fragrant repelling foulness (Fangxiang Pihui)” is considered to be a therapeutic strategy against epidemic since the Ming Dynasty. In TCM, many fragrant herbs can be used for the prevention and treatment of epidemics through burning, smelling, sneezing, and bathing [14]. LBO is commonly used to treat cold, cough, headache, sore throat, swelling, and pain. However, due to the complex composition, the bioactivity investigation and mechanism study of LBO is very limited. In this research, we evaluate *in vitro* and *in vivo* anti-influenza effects of LBO and investigate the underlying mechanisms.

## 2. Material and Methods

### 2.1. Preparation of LBO

LBO (Lot No. 20E201) was provided by Guangdong Luofushan Sinopharm Co., Ltd. (Huizhou, China). LBO is composed of methyl salicylate, peppermint oil, camphor oil, turpentine oil, eucalyptus oil, camphor, menthol, cinnamon oil, *Ocimum gratissimum* oil, borneol, star anise oil, and Baicaojing extraction (the rest of sixty-eight herbs, listed in Table S1, were extracted with 5-fold tea seed oil for 15 days in room temperature, and the solution was filtered to get baicaojing extraction). Methyl salicylate 250 g, peppermint oil 250 g, camphor oil 150 g, turpentine oil 95 g, eucalyptus oil 40 g, camphor 30 g, menthol 27.5 g, cinnamon oil 20 g, *Ocimum gratissimum* oil 15 g, borneol 2.5 g, and star anise oil 2g were mixed with the baicaojing extraction, with extra tea seed oil added to get 1 liter of LBO (relative density 0.96 mg/mL).

### 2.2. Drugs and Reagents

Reference substance: methyl salicylate, camphor, trans-anethole, eugenol, cinnamaldehyde, eucalyptol, borneol, menthol, nitidine chloride, ligustilide, ginsenoside rb1, hesperidin, and kinsenoside were purchased from National Institutes for Food and Drug Control (Beijing, China).

Ribavirin was purchased from Aladdin (Shanghai, China). Polyinosinic-polycytidylic acid (Poly I: C), methyl thiazolyl tetrazolium (MTT), and 2′-(4-methylumbelliferyl)-*α*-D-N-acetylneuraminic acid sodium salt hydrate (MU-NANA) were purchased from Sigma-Aldrich (Missouri, USA). Lipo3000 was purchased from Invitrogen (Massachusetts, USA). Mouse interleukin-6 (IL-6) enzyme linked immunosorbent assay (ELISA) kit and mouse interleukin-1*β* (IL-1*β*) ELISA kit were purchased from Shanghai Enzyme-linked Biotechnology (Shanghai, China). TRIzol reagent was purchased from Invitrogen (Massachusetts, USA). PrimeScript RT-PCR Kit was purchased from Takara (Beijing, China). RIPA lysis buffer was purchased from Beyotime (Shanghai, China).

Anti-NF-*κ*B p65 (phospho S536) antibody (ab86299) was provided by Abcam (Cambridge, UK). Anti-NF-*κ*B p65 antibody (#8242), anti-phospho-IRF-3 (Ser396) antibody (#29047), and anti-IRF-3 antibody (#4302) were provided by Cell Signaling Technology Inc. (Massachusetts, USA). IVA NP (nucleoprotein) antibody (GTX125989), IVA PB2 protein antibody (GTX125926), and IVA M2 (matrix protein) antibody (GTX125951) were provided by Gene Tex (California, USA). Anti-GAPDH antibody 60004-1-Ig was provided by Proteintech (Wuhan, China).

### 2.3. Viruses

Influenza virus A/FM/1/47 (H1N1) and influenza virus A/WSN/1933 (H1N1) were donated by Prof. Zifeng Yang in the State Key Laboratory of Respiratory Diseases, Guangzhou Medical University (Guangzhou, China). *In vivo* influenza virus experiment was performed in BSL2 barrier animal facility in Guangzhou Medical University. *In vitro* influenza virus experiment was performed in BSL2 laboratory in Southern Medical University.

### 2.4. GC-MS and LC-MS Analysis of LBO

The compositions of LBO were analyzed using GC-MS and LC-MS methods. GC-MS analysis was performed on an Agilent 7890B GC system coupled to an Agilent 7000D triple quadrupole MS (QQQ-MS) (Agilent Technologies, California, USA) and HP-INNOWAX column (30 m × 0.32 mm × 0.25 *μ*m, Agilent Technologies, California, USA). The column temperature was programmed as follows: the initial temperature was 50°C and increased to 90°C at 10°C/min, then raised to 150°C at 15°C/min and held for 5 min, and finally increased to 240°C at 20°C/min and held for 5 min. Mass scanning was examined in ionization mode of EI at 45–500 amu.

LC-MS analysis was performed on Vanquish UHPLC system coupled to Orbitrap Fusion Tribrid mass spectrometer (Thermo Fisher Scientific, Massachusetts, USA) and Hypersil Gold column (100 mm × 2.1 mm × 1.9 *μ*m, Thermo Fisher Scientific, Massachusetts, USA). The elution was performed with mobile phase composed of 0.1% formic acid in water (solvent A) and acetonitrile (solvent B) using the following gradient program at the ﬂow rate of 0.3 mL/min: 0–7 min, linear gradient 22–40% (B); 7–12 min, linear gradient 40–70% (B); 12–22 min, linear gradient 70–50% (B); 22–23 min, linear gradient 50–22% (B); 23–28 min, and isocratic gradient 22–22% (B). The column temperature was 35°C and the injection volume was 2 *μ*L. Mass scanning was examined in ionization mode of ESI at scan range (*m/z*) of 120–1200.

### 2.5. Cytotoxicity Assay

Madin–Darby Canine Kidney (MDCK) cells were cultured in Dulbecco's modified Eagle's medium with 10% fetal bovine serum, 100 units/mL penicillin, and 100 *μ*g/mL streptomycin. The MDCK cells are recommended by ATCC to using as the host cells of influenza [15]. MDCK cells were plated in 96-well plates and cultured overnight at 37°C in 5% CO_2_. The LBO were mixed with tween 20 and diluted with DMEM. The medium was removed and the cells were then incubated with various concentrations of LBO (800–50 *μ*g/mL) for 48 h. MTT was added to each well and further incubated for 4 h. The medium was subsequently removed, and formazan crystals were solubilized with dimethyl sulfoxide (DMSO). The absorbance was tested at 490 nm.

### 2.6. Antiviral Assay

MDCK cells were infected with influenza virus A/WSN/1933 (H1N1) at 37°C for 2 h. The medium was replaced with various concentrations of LBO (12.5–100 *μ*g/mL) at 37°C in 5% CO_2_ for 2 days. MTT was added to each well and further incubated for 4 h. The medium was subsequently removed, and formazan crystals were solubilized with DMSO and tested at 490 nm.

MDCK cells were infected with influenza virus A/WSN/1933 (H1N1) at 37 °C for 2 h. The medium was replaced with LBO (50 *μ*g/mL, 100 *μ*g/mL) at 37°C in 5% CO_2_ for 24 h. MDCK cells were harvested for RT-PCR analysis.

### 2.7. Plaque Reduction Assay

Confluent monolayers of MDCK cells were infected with influenza virus A/WSN/1933 (H1N1) at 37°C for 2 h. After incubation, the cell monolayer was covered with the overlay medium containing LBO (50 *μ*g/mL and 100 *μ*g/mL) and further cultured at 37°C in 5% CO_2_ for 72 h. Subsequently, the overlay medium was removed, and the cell monolayer was fixed with 4% paraformaldehyde and stained with 1% crystal violet, and the plaques were visualized with ImmunoSpot S6 (CTL, Ohio, USA).

### 2.8. Immunofluorescence Staining

MDCK cells were infected with influenza virus A/WSN/1933 (H1N1) at 37°C for 2 h. After incubation, the medium was replaced with LBO (50 *μ*g/mL and 100 *μ*g/mL) and incubation at 37°C in 5% CO_2_ for another 24 h; the cells were fixed with 4% paraformaldehyde for 30 min and then permeabilized with 0.1% Triton X-100 for 5 min. After blocking with 3% BSA for 1 h, the cells were incubated with anti-IVA NP antibody overnight at 4 °C. The cells were incubated with FITC-labeled secondary antibody for 1 h and further stained with 4′',6-diamidino-2-phenylindole (DAPI). The fluorescence was visualized using Axio Observer (Zeiss, Oberkochen, Germany).

### 2.9. NA Inhibition Assay

First, 60 *μ*l of influenza virus A/WSN/1933 (H1N1) was incubated with 10 *μ*l of LBO (200–3.12 *μ*g/mL) and zanamivir at 37°C for 10 min in black 96-well microplate. Next, 30 *μ*l of MU-NANA (80 *μ*M) was added and incubated at 37°C for 30 min. Fluorescence was measured at Ex = 355 nm and Em = 460 nm.

### 2.10. Hemagglutination Inhibition Assay

50 *μ*L of hemagglutinin (HA) was mixed with an equal volume of LBO (200–3.12 *μ*g/mL) or HA antibody and incubated for 30 min at 4°C. Afterward, 50 *μ*L of 1% chicken RBCs was added to each well and incubated at 37°C for 40 min to test the agglutination.

### 2.11. Time-of-Addition Assay

MDCK cells were infected with the influenza virus A/WSN/1933 (H1N1) for 1 h. After removing unabsorbed virus, the cells were treated with LBO (100 *μ*g/mL) at indicated time intervals (0–2, 2–5, 5–8, 8–10, and 0–10 h), which covered one cycle of influenza virus replication. At 10 h p.i., the expression level of viral NP protein was determined by western blotting.

### 2.12. Poly I:C Treatment

The Poly I:C is a synthetic analog of viral double-stranded RNA. A549 cell line is human lung cells. The Poly I:C-treated A549 cells were employed to investigate the anti-influenza mechanism of LBO, specifically, the influence of LBO on the host immunity during the virus infection [11]. A549 cells were plated in 6-well plates (4 × 10^5^ cells/well) and incubated overnight. The medium was replaced with various concentrations of LBO (50 *μ*g/mL and 100 *μ*g/mL); cells were then transfected with Poly I:C (Lipo3000 regent treated) at 37°C in 5% CO_2_ for 24 h. A549 cells were harvested for RT-PCR and western blot analysis.

### 2.13. Animals and Treatment

Female BALB/c mice (16–18 g) were purchased from Guangdong Medical Laboratory Animal Center (Guangzhou, China). The animal experiments were performed to the Guidelines of Guangdong Regulation for the Administration of Laboratory Animals. This animal study was approved by the Ethics Committee of Guangzhou Medical University (2019–645). The mice were intranasally inoculated with 35 *μ*L of influenza virus A/FM/1/47 (H1N1). After the infection, mice were treated with LBO (100 *μ*g/mL and 400 *μ*g/mL) with YLS-8B animal atomization device platform (Yiyan, Jinan, China). According to the instructions, 80% of aerosol diameter was 1–5 *μ*m. The mice were treated in the container (285 × 240 × 160 mm), the device speed was set as 1.5 *μ*L/s, each treatment lasted for 30 min, and LBO was administered twice a day for 5 days. The control animals were treated with the solvent. Ribavirin was used as a positive control.

Five days after virus infection, mice were weighed and sacrificed. The lung tissues were removed and weighed. The lung index was calculated (lung index = lung weight/body weight × 100%). Lung tissue was harvested for histopathologic examination and western blotting analysis.

### 2.14. RT-PCR

MDCK cells and A549 cells were scraped from the plate, and the total RNA of the cells were extracted with TRIzol reagent. The quality of the extracted RNA was determined by optical density measurement at 260 nm on a spectrophotometer (Thermo Fisher Scientific, Massachusetts, USA). Retrotranscription was performed using 2 *μ*g of RNA. The mRNA levels of the target genes were determined by PrimeScript RT-PCR Kit using LightCycler 480 (Roche, Basel, Switzerland). Primer sequences are given in Table S2; the expression of target genes was tested by 2^−ΔΔCt^ method.

### 2.15. Immunoblotting

The protein of A549 and lung tissue were extracted with RIPA lysis buffer. Equal amounts of protein were separated using SDS-PAGE and transferred onto PVDF membranes. Membranes were blocked and incubated overnight at 4°C with primary antibodies (p-P65, P65, p-IRF3, IRF3, NP, M2, and PB2 GAPDH) and secondary HRP conjugated antibody. Western blot bands were examined by FluorChem E (Protein Simple, California, USA).

### 2.16. Enzyme-Linked Immunosorbent Assay (ELISA)

The levels of IL-1*β* and IL-6 were determined by ELISA kit (Meilian, Shanghai, China) according to the manufacturer's instructions. The absorbance of each well was tested at 450 nm.

### 2.17. Histopathological Analysis

The lung tissues were harvested on the 6th day after infection and fixed with 4% paraformaldehyde for 24 h and embedded in paraffin. Embedded lung tissues were cut into 5 *μ*m thick sections. The sections were stained with hematoxylin and eosin (H&E). The sections were captured by Axio Observer (Zeiss, Oberkochen, Germany).

### 2.18. Statistical Analyses

The data were expressed as means ± SEM and performed using GraphPad Prism v.6 (GraphPad Software). Statistical comparisons of the data were analyzed with one-way ANOVA followed by Dunnett's post hoc test. A value of *p* < 0.05 was considered to be significant.

## 3. Results

### 3.1. GC-MS and LC-MS Analysis of LBO

**Figure 1:**
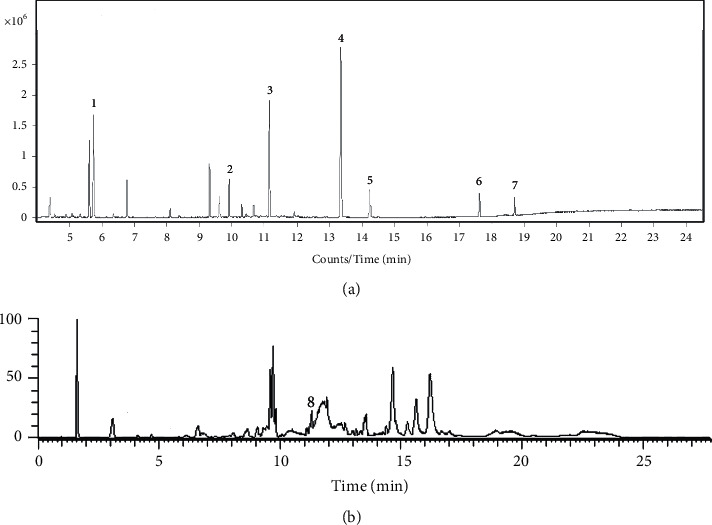
GC-MS and LC-MS analysis of LBO. (a) The GC-MS chromatogram of volatile part of LBO, (1) eucalyptol, (2) camphor, (3) menthol, (4) methyl salicylate, (5) trans-anethole, (6) cinnamaldehyde, and (7) eugenol. (b) The LC-MS chromatogram of nonvolatile part of LBO, (8) ligustilide.

The GC-MS and LC-MS analysis of LBO is shown in Figure 1. Thirteen reference substances were used to identify the compositions in LBO. Seven components (eucalyptol, camphor, menthol, methyl salicylate, trans-anethole, cinnamaldehyde, and eugenol) were identified based on the retention times and product ion by GC-MS (Figures 1(a) and S1(a)). Ligustilide was identified based on the retention times and product ion by LC-MS (Figures 1(b) and S1(b)).

### 3.2. The *In Vitro* Anti-Influenza Activity of LBO

**Figure 2:**
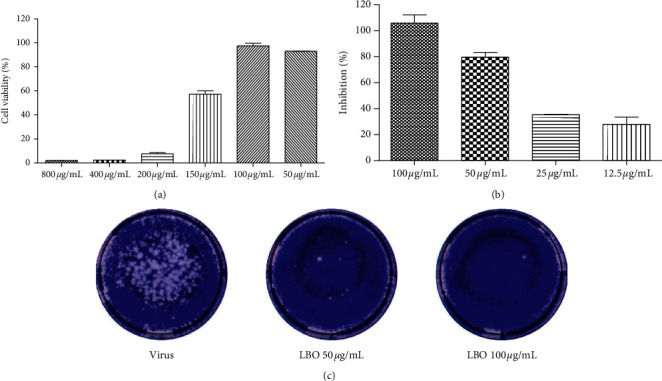
Anti-influenza activity of LBO. (a) The cytotoxicity of LBO in MDCK cells. (b) The anti-influenza activity of LBO. (c) The plaque reduction of LBO against influenza viruses.

To investigate the antivirus activity of LBO, we evaluated the influence of LBO on the cell viability of MDCK. At a concentration of 100 *μ*g/mL or less, LBO exerted no significant cytotoxicity in MDCK cells (Figure 2(a)). In MTT assay, LBO (25–100 *μ*g/mL) could inhibit the reduction of cell viability induced by the influenza virus in a dose-dependent manner (Figure 2(b)). The infection of influenza virus A/WSN/1933 (H1N1) could induce plaque formation in the MDCK infection model. LBO (50 *μ*g/mL and 100 *μ*g/mL) could effectively decrease the plaque number (Figure 2(c)).

### 3.3. The Influence of LBO on the NP Expression in MDCK Cells

**Figure 3:**
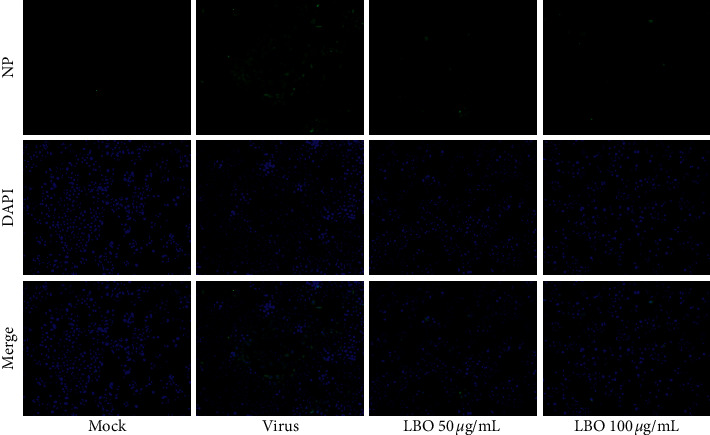
The influence of LBO on the NP expression in MDCK cells. The inﬂuenza A virus NP was stained with FITC-labeled antibody (green). The cell nuclei were stained with DAPI (blue). Samples were captured with a ﬂuorescent microscope (magnification: 200×).

The expression and location of NP were used to confirm the influence of LBO on virus replication and nuclear export of viral ribonucleoprotein (vRNP). As shown in Figure 3, with the infection of influenza virus A/WSN/1933 (H1N1), a strong immunofluorescence signal, as well as the export of vRNP, could be observed in MDCK cells. LBO (50 *μ*g/mL and 100 *μ*g/mL) could remarkably downregulate the NP expression, while the vRNP export could not be alleviated by the treatment of LBO.

### 3.4. The Influence of LBO on the Replication Cycle of Influenza Virus

The neuraminidase inhibition test, hemagglutination inhibition test, and viral replication test were performed to investigate the effects of LBO on the influenza virus. As shown in Figure S2, LBO could not inhibit the neuraminidase and hemagglutination activity. During the whole replication cycle of influenza virus A/WSN/1933 (H1N1), LBO could not downregulate the expression of NP protein. These results indicated that LBO may not directly interact with IVA.

### 3.5. The Influence of LBO on the mRNA Expressions of IL-1*β*, IL-6, and IFN-*β* in Virus-Infected MDCK Cells and Poly I:C-Treated A549 Cells

**Figure 4:**
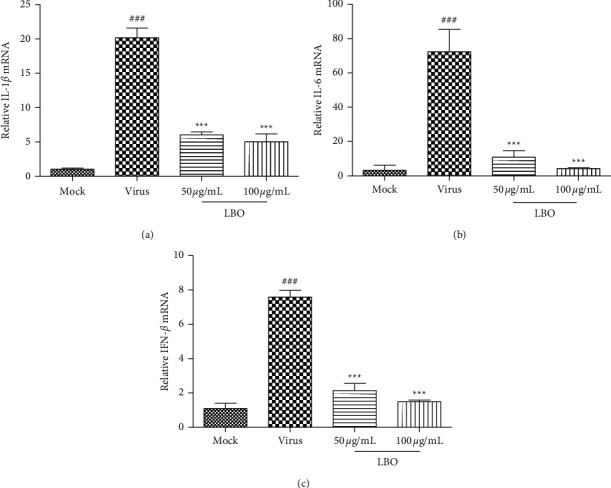
The influence of LBO on the mRNA expressions of IL-1*β*, IL-6, and IFN-*β* in IVA-infected MDCK cells. (a) The influence of LBO on the mRNA expressions of IL-1*β*. (b) The influence of LBO on the mRNA expressions of IL-6. (c) The influence of LBO on the mRNA expressions of IFN-*β*. Compared with mock group: ^###^*p* < 0.001; compared with virus group: ^*∗∗∗*^*p* < 0.001.

**Figure 5:**
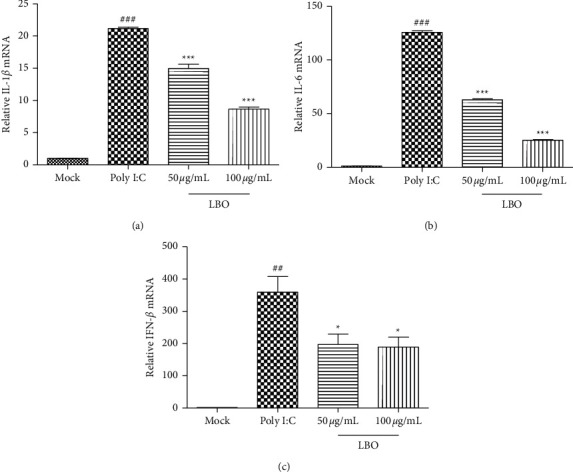
The influence of LBO on the mRNA expressions of IL-1*β*, IL-6, and IFN-*β* in Poly I:C-stimulated A549 cells. (a) The influence of LBO on the mRNA expressions of IL-1*β*. (b) The influence of LBO on the mRNA expressions of IL-6. (c) The influence of LBO on the mRNA expressions of IFN-*β*. Compared with mock group: ^###^*p* < 0.001, ^##^*p* < 0.01; compared with virus group: ^*∗∗∗*^*p* < 0.001, ^*∗*^*p* < 0.05.

The expressions of IL-1*β*, IL-6, and IFN-*β* were tested to evaluate the influence of LBO on the cytokine. With the stimulation of the influenza virus A/WSN/1933 (H1N1) as well as the Poly I:C, the mRNA expressions of IL-1*β*, IL-6, and IFN-*β* in MDCK cells and A549 cells could be strongly upregulated. With the treatment of LBO, the expression of IL-1*β*, IL-6, and IFN-*β* could be remarkably inhibited (Figures 4 and 5).

### 3.6. The Influence of LBO on the Levels of NF-*κ*B P65 and Interferon Regulatory Factor 3 in Poly I:C-Treated A549 Cells

**Figure 6:**
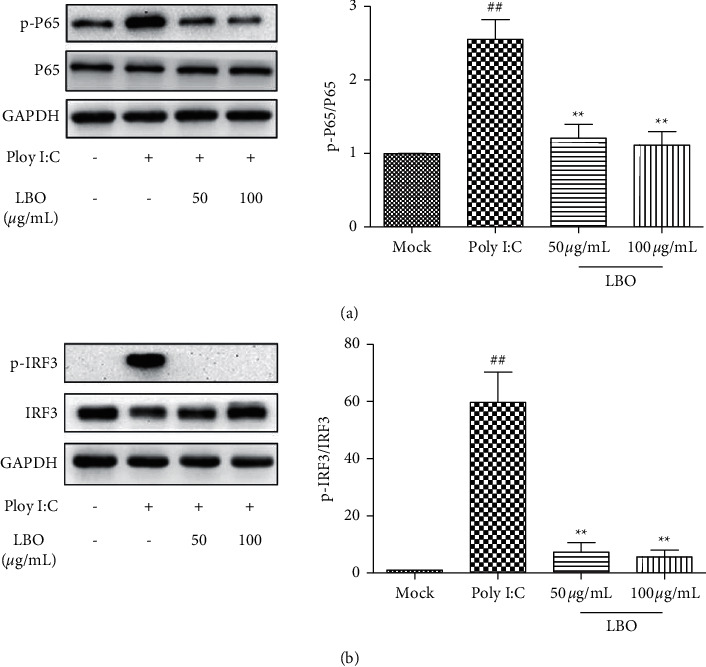
The influence of LBO on the levels of P65 and IRF3 in Poly I:C-stimulated A549 cells. (a) The influence of LBO on the expression of P65. (b) The influence of LBO on the expression of IRF3. Compared with mock group: ^##^*p* < 0.01; compared with virus group: ^*∗∗*^*p* < 0.01.

To investigate the underlying mechanisms of the inhibition of LBO on cytokine expression, we tested the phosphorylation and expression levels of NF-*κ*B P65 (P65) and interferon regulatory factor 3 (IRF3) in Poly I:C-treated A549 cells (Figure 6). With the stimulation of Poly I:C, the phosphorylation of P65 and IRF3 was strongly increased. LBO treatment could significantly alleviate the activation of P65 and IRF3 in A549 cells.

### 3.7. The Anti-Influenza Activity of LBO in Mice

**Figure 7:**
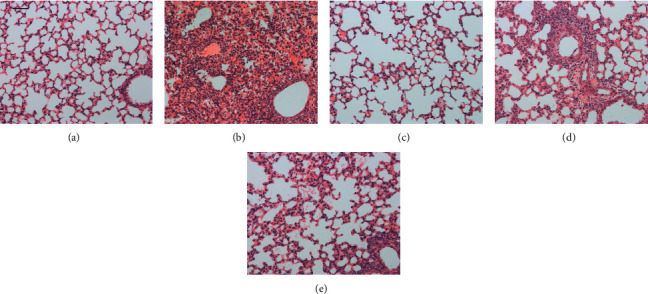
The influence of LBO on the pathological changes in the lung tissue in IVA-infected mice. (a) Control group, (b) virus group, (c) ribavirin 75 mg/kg group, (d) LBO 100 *μ*g/mL group, and (e) LBO 400 *μ*g/mL group; ribavirin was used as a positive control (magnification: 200×, scale bar = 100 *μ*m).

Five days after virus infection, the histopathologic changes in the lung tissues were determined, and the lung index was calculated. With the infection of influenza virus A/FM/1/47 (H1N1), severe alveolar thickening and inflammatory cell infiltration could be observed in the lung tissues; the lung index was significantly increased from 0.7896% to 1.2952%. Inhalation of LBO (100 *μ*g/mL and 400 *μ*g/mL) with atomizer could significantly alleviate the pulmonary inflammation and the lung index; the inhibition rates were 29.23% and 33.03% (Table 1 and Figure 7).

### 3.8. The Influence of LBO on the Serum Levels of IL-1*β* and IL-6 in Mice

**Figure 8:**
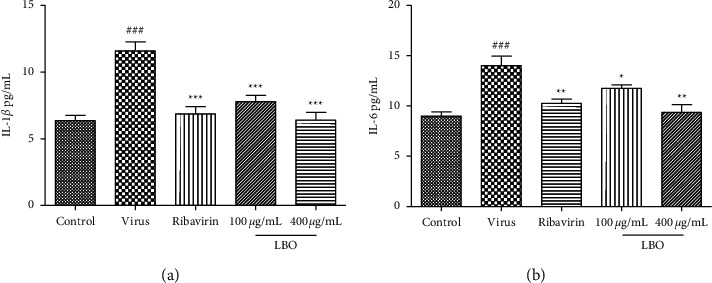
The influence of LBO on the serum levels of IL-1*β* and IL-6 in IVA-infected mice. (a) The influence of LBO on the levels of IL-1*β*. (b) The influence of LBO on the levels of IL-6. Compared with control group: ^###^*p* < 0.001; compared with virus group: ^*∗∗∗*^*p* < 0.001, , ^*∗∗*^*p* < 0.01, ^*∗*^*p* < 0.05. Ribavirin was used as a positive control.

We further evaluate the effects of LBO on the expression of proinflammatory cytokines in mice serum. The concentrations of IL-1*β* and IL-6 in serum were tested with ELISA. The influenza virus A/FM/1/47 (H1N1) infection could substantially upregulate the serum levels of IL-1*β* and IL-6. The treatment of LBO could significantly reduce the expression of IL-1*β* and IL-6 in mice (Figure 8).

### 3.9. The Influence of LBO on the Virus Proteins, P65 and IRF3, in Mice

**Figure 9:**
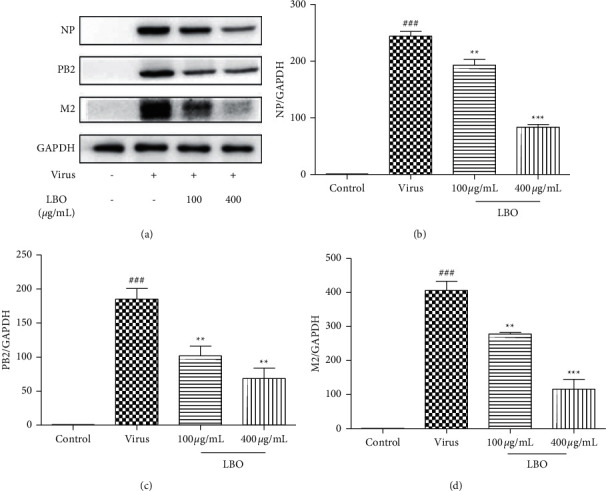
The influence of LBO on the levels of NP, PB2, and M2 in the lung tissue of IVA-infected mice. (a) Western blot result of NP, PB2, and M2. (b) Quantitative analysis of NP. (c) Quantitative analysis of PB2. (d) Quantitative analysis of M2. Compared with control group: ^###^*p* < 0.001; compared with virus group: ^*∗∗∗*^*p* < 0.001, ^*∗∗*^*p* < 0.01.

**Figure 10:**
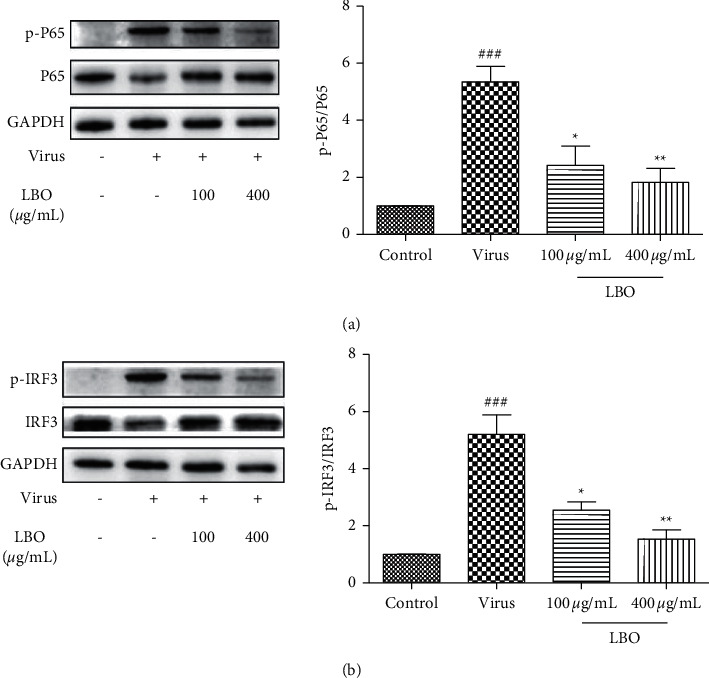
The influence of LBO on the levels of P65 and IRF3 in the lung tissue of IVA-infected mice. (a) The influence of LBO on the expression of P65. (b) The influence of LBO on the expression of IRF3. Compared with mock group: ^###^*p* < 0.001; compared with virus group: ^*∗∗∗*^*p* < 0.001, ^*∗*^*p* < 0.05.

Nucleoprotein (NP), PB2, and matrix 2 ion channel (M2) are the three important proteins of IVA. We tested the expression of NP, PB2, and M2 to evaluate the virus clearance effect of LBO in the lung tissue. With the application of LBO, the expression of NP, PB2, and M2 could be significantly reduced (Figure 9). To further understand the underlying mechanisms of the treatment of LBO on the pneumonia mice, the activation of P65 and IRF3 was examined. After being infected by the influenza virus A/FM/1/47 (H1N1), the P65 and IRF3 in lung tissue were remarkably activated. Inhalation of LBO for five days could strongly inhibit the activation of P65 and IRF3 in mice (Figure 10).

## 4. Discussion

According to the TCM theory of “fragrant repelling foulness “(Fangxiang Pihui),” burning, smelling, sneezing, and bathing the fragrant herbs could be used for the prevention and treatment of epidemic [14]. Many modern medical investigations support this ancient theory. According to the team of Zhang Q, the inhalation of the sachet (putting fragrant herbs into the small pocket) may prevent influenza through the enhancement of innate immune responses in mice [16]. Li Y proved that inhalation of Bing-Xiang-San, a fragrant Chinese formula, with an atomizer could inhibit the IVA infection in mice model [17]. Fragrant herbs are commonly rich in essential oil; many investigations indicated that essential oil could inhibit the infection of influenza both *in vitro* and *in vivo* [15, 18, 19].

The prescription of LBO is complex, but the content of essential oil and related components is more than ninety percent (882 g/L or 91.87%). Thirteen reference compounds were used to identify the composition of LBO. With the GC-MS method and LC-MS method, eight compounds (eucalyptol, camphor, menthol, methyl salicylate, trans-anethole, cinnamaldehyde, eugenol, and ligustilide) were identified. The bioactive compounds in LBO are uncertain; however, according to other researches, cinnamon oil, eugenol, and eucalyptol have anti-influenza effect. Inhalation and nasal inoculation of cinnamaldehyde, the main component of cinnamon oil, could significantly increase the survival rates of IVA mice [20]. Eugenol, the major component *Ocimum gratissimum* oil, exerted anti-influenza activity in both liquid and vapor phases via inhibition of ERK, p38, and IKK signal pathways [19, 21]. Eucalyptol from eucalyptus oil was able to protect against IVA infection in mice through the attenuation of pulmonary inflammatory responses [22]. Moreover, trans-anethole from star anise oil could reduce LPS-induced acute lung injury by resolution of pulmonary inflammation [23].

In this research, we demonstrated that LBO significantly inhibits the infection of IVA, decreases the formation of plaques, and reduces the expression of viral nucleoprotein. To explore the mechanism of anti-influenza effects, we examined the neuraminidase inhibition and hemagglutination inhibition as well as viral replication inhibition effects of LBO. However, the present results indicate that LBO could not affect the IVA directly. Many investigations about the anti-influenza effects of herbal formulas found that apart from the direct antivirus effects these herbal formulas could regulate the immune response caused by the viral infection [24, 25]. Hence, we hypothesized that the anti-influenza effects of LBO may result from the regulation of host immunity.

After the infection of airway epithelial cells, the influenza virus can be recognized by TLR3 and result in the activation of NF-*κ*B and IRF3 [9, 11]. The activation of NF-*κ*B leads to the gene transcription of proinflammatory cytokines. The high levels of IL-1*β* and IL-6, typical cytokines that contribute to the cytokine storm phenomenon, are proved to be correlated directly with tissue injury [26]. Many findings proved that NF-*κ*B pathway is a prerequisite for IVA infection, and the inactivation of NF-*κ*B pathway can protect the mice from infection [27, 28]. Our research found that LBO could reduce the expression of IL-1*β* and IL-6 through the suppression of P65 phosphorylation.

The activation of IRF3 leads to the expression of type I IFNs, IFN-*α*, and IFN-*β*, which contribute to the restriction of viral replication [29]. However, excessive type I IFNs in response to acute influenza infection contribute to immune cell-mediated tissue damage [30, 31]. Interestingly, when it comes to the activation of IRF3 and transcription of IFN-*β*, host cells show different responses to different subtypes of IVA [32]. In this research, we found that influenza virus A/FM/1/47 (H1N1) infection and Poly I:C contribute to the activation of IRF3. Treatment with LBO blocked the activation of IRF3 and inhibited the expression of IFN-*β*.

The different concentrations of LBO were used in cell culture and animal study. The concentrations of LBO in the cell culture study were designed according to the cytotoxicity of LBO. The concentrations of LBO in the animal study were designed according to the results of cell culture study and our *in vivo* pre-experiment. Moreover, LBO exert more anti-inflammatory effects in MDCK cells compared to Poly I:C-stimulated A549 cells (Figures 4 and 5). The different anti-inflammatory effects of LBO in two models may result from the different systems.

The LBO is an essential oil-rich formula, the anti-influenza effects of which are considered to be contributed by the volatile components according to the TCM theory and modern researches [19, 33]. Oral administration of LBO is excluded considering about the traditional application and the great amount of volatile components. The administration of LBO was designed according to its clinical use, the inhalation of which could be used for the treatment of cough and sore throat. Inhalation with atomizer is employed by many researchers to investigate the essential oil related product [17, 20]. With the animal atomization device platform, we could control the dosage through the control of LBO concentration, device speed, and the treatment time.

In conclusion, the present study demonstrated that LBO could effectively prevent IVA infection both *in vitro* and *in vivo*. The anti-influenza effects of LBO may be attributed to the reduction of proinflammatory cytokines and the blocking of P65 and IRF3 activation. These findings suggest that LBO can be developed as an alternative therapeutic agent for influenza prevention.

## Figures and Tables

**Table 1:** The influence of LBO on the lung index in influenza-infected mice.

Group	Dose	Lung index (%)	Inhibition rate (%)
Control	—	0.7896	—
Virus	—	1.2952^###^	—
Ribavirin	75 mg/kg	0.9085^*∗∗*^	76.49
LBO	100 *μ*g/mL	1.1474^*∗∗*^	29.23
LBO	400 *μ*g/mL	1.1282^*∗∗*^	33.03

Compared with control group^###^*p* < 0.001; compared with virus group ^*∗∗*^*p* < 0.01. Ribavirin was used as a positive control.

## Data Availability

All data generated or analyzed during this study are included in this published article.

## Supplementary Materials

Supplementary Materials Table S1: the compositions of LBO. Table S2: the primer sequences. Figure S1: GC-MS and LC-MS chromatogram of reference substances. Figure S2: the influences of LBO on the influenza virus.
