# Supplementary material for: Essential Oil-Rich Chinese Formula Luofushan-Baicao Oil Inhibits the Infection of Influenza A Virus through the Regulation of NF-κB P65 and IRF3 Activation
Source: Evid Based Complement Alternat Med. 2021 Aug 30;2021:5547424. doi: 10.1155/2021/5547424 (PMC8421167; doi:10.1155/2021/5547424)
Supplement: Supplementary Materials — Table S1: the compositions of LBO. Table S2: the primer sequences. Figure S1: GC-MS and LC-MS chromatogram of reference substances. Figure S2: the influences of LBO on the influenza virus. [file 5547424.f1.doc]

**Supplementary materials**

Table S1 The compositions of LBO

| **Chinese name** | **Compound/Botanical name** | **Parts used** | **Concentrate** |
| --- | --- | --- | --- |
| Shui yang suan jia zhi | Methyl salicylate | - | 250 g/L |
| Bo he su you | Peppermint oil | - | 250 g/L |
| Zhang you | Camphor oil | - | 150 g/L |
| Song jie you | Turpentine oil | - | 95 g/L |
| An you | Eucalyptus oil | - | 40 g/L |
| Zhang nao | Camphor | - | 30 g/L |
| Bo he nao | Menthol | - | 27.5 g/L |
| Rou gui you | Cinnamon oil | - | 20 g/L |
| Ding xiang luo le you | Ocimum gratissimum oil | - | 15 g/L |
| Bing pian | Borneol | - | 2.5 g/L |
| Ba jiao hui xiang you | Star anise oil | - | 2 g/L |
| Liang mian zhen | *Zanthoxylum nitidum* | Root | 1 g/L |
| Xu chang qin | *Cynanchum paniculatum* | Root and rhizome | 1 g/L |
| Jiu li xiang | *Murraya exotica* | Leaf and stem | 1 g/L |
| Xin yi | *Magnolia biondii* | Flower | 1 g/L |
| Hong hua | *Carthamus tinctorius* | Flower | 1 g/L |
| Shui fu rong | *Limnophila aromatica* | Whole plant | 1 g/L |
| Juan bai | *Selaginella tamariscina* | Whole plant | 1 g/L |
| Jin bu huan | *Stephania epigaea* | Root | 1 g/L |
| Qian li guang | *Senecio scandens* | Leaf and stem | 1 g/L |
| Da tou chen | *Adenosma indianum* | Whole plant | 1 g/L |
| Dang gui | *Angelica sinensis* | Root | 1 g/L |
| E bu shi cao | *Centipeda minima* | Whole plant | 1 g/L |
| San qi | *Panax notoginseng* | Root and rhizome | 1 g/L |
| Zhong jie feng | *Sarcandra glabra* | Whole plant | 1 g/L |
| Ji gu xiang | *Croton crassifolius* | Root | 1 g/L |
| Sha ren | *Amomum villosum* | Root | 0.55 g/L |
| Du huo | *Angelica pubescens* | Root | 0.55 g/L |
| Qiang huo | *Notopterygium inchum* | Root and rhizome | 0.55 g/L |
| Jiang pi | *Zingiber officinale* | Rhizome peel | 0.55 g/L |
| Chen pi | *Citrus reticulata* | Fruit peel | 0.55 g/L |
| Xiang fu | *Cyperus rotundus* | Rhizome | 0.5 g/L |
| Ye ju hua | *Ghrysanthemum indicum* | Flower | 0.5 g/L |
| Shan bai zhi | *Inula cappa* | Root and rhizome | 0.5 g/L |
| Gui zhi | *Cinnamomum cassia* | Stem | 0.5 g/L |
| Xiao luo san | *Ardisia punctata* | Root | 0.5 g/L |
| Man jing zi | *Vitex trifolia* | Fruit | 0.5 g/L |
| Jie geng | *Platycodon grandiflorum* | Root | 0.5 g/L |
| Zi zhu ye | *Callicarpa formosana* | Leaf | 0.5 g/L |
| Di dan cao | *Elephantopus scaber* | Whole plant | 0.5 g/L |
| Xi xin | *Asarum sieboldii* | Root and rhizome | 0.5 g/L |
| Wu zhi gan | *Citrus medica* | Fruit | 0.5 g/L |
| Rou dou kou | *Myristica fragrans* | Seed | 0.5 g/L |
| Mu fang ji | *Cocculus trilobus* | Root | 0.5 g/L |
| San cha ku | *Evodia lepta* | Leaf and stem | 0.5 g/L |
| Shan yin hua | *Lonicera confusa* | Flower | 0.5 g/L |
| Jiu bi ying | *Ilex rotunda* | Bark | 0.5 g/L |
| Bai ban feng he | *Dendropanax dentiger* | Root and stem | 0.5 g/L |
| Bi cheng qie | *Litsea cubeba* | Fruit | 0.5 g/L |
| Ma huang | *Ephedra sinica* | Bark | 0.5 g/L |
| Di ren | *Melastoma dodecan* | Whole plant | 0.5 g/L |
| Fang feng | *Saposhnikovia divaricata* | Root | 0.5 g/L |
| Ban zhi lian | *Scutellaria barbata* | Whole plant | 0.5 g/L |
| Tie bao jin | *Berchemia lineata* | Root | 0.5 g/L |
| Chai hu | *Bupleurum scorzonerifolium* | Root | 0.5 g/L |
| Fei tian qin lao | *Alsophila spinulosa* | Stem | 0.5 g/L |
| Ji gu cao | *Abrus cantoniensis* | Whole plant | 0.5 g/L |
| Jing jie | *Schizonepeta tenuifolia* | Leaf and stem | 0.5 g/L |
| Hu zhang | *Polygonum cuspidatum* | Root and rhizome | 0.5 g/L |
| Gou teng | *Uncaria rhynchophylla* | Stem | 0.5 g/L |
| Yi zhi huang hua | *Solidago decurrens* | Whole plant | 0.45 g/L |
| Bai hua deng long | *Clerodendron fortunatum* | Leaf and stem | 0.43 g/L |
| Bai hua she she cao | *Hedyotis diffusa* | Whole plant | 0.43 g/L |
| Ren zi cao | *Kummerowia striata* | Whole plant | 0.25 g/L |
| Jin xian feng | *Anoectochilus roxburghii* | Whole plant | 0.25 g/L |
| Shi xian tao | *Pholidota chinensis* | Whole plant | 0.25 g/L |
| Wu yue ai | *Artemisia indica* | Whole plant | 0.25 g/L |
| Zao jiao ci | *Gleditsia sinensis* | Spina | 0.25 g/L |
| Mu xiang | *Aucklandia lappa* | Root | 0.25 g/L |
| Shan zhi ma | *Helictercs angustifolia* | Root | 0.25 g/L |
| Yi mu cao | *Leonurus japonicus* | Whole plant | 0.25 g/L |
| Zi su ye | *Perilla frutescens* | Leaf | 0.25 g/L |
| Dao kou cao | *Achyranthes aspera* | Whole plant | 0.25 g/L |
| Ce bai ye | *Platycladus orientalis* | Leaf and stem | 0.25 g/L |
| Jin er huan | *Asarum insigne* | Whole plant | 0.25 g/L |
| Yi duo yun | *Pittosporum glabratum* | Leaf | 0.25 g/L |
| Chong lou | *Paris polyphylla* | Rhizome | 0.25 g/L |
| Yu xing cao | *Houttuynia cordata* | Leaf and stem | 0.25 g/L |
| Diao huang | *Polygala fallax* | Root | 0.25 g/L |

Table S2 The primer sequences

| **Primer name** | **Source** | **Forward primer** | **Reverse primer** |
| --- | --- | --- | --- |
| IL-1β | *Canis lupus* | CCTGTGGTCTTGGGCATCAA | TCTAGCTGTAGGGTGGGCTT |
| IL-6 |  | GGCTACTGCTTTCCCTACCC | CAGTGCAGAGATTTTGCCGAG |
| IFN-β |  | AACAGGACTCTTCTGCACCTG | ATGCTGTACTCCTTGGCCTTC |
| GAPDH |  | CCCACTCTTCCACCTTCGAC | TGGTCCAGGAGGCTCTTACT |
| IL-1β | *Homo sapiens* | CCTGAGCTCGCCAGTGAAA | GTGGTGGTCGGAGATTCGTA |
| IL-6 |  | TAGTGAGGAACAAGCCAGAGC | GTTGGGTCAGGGGTGGTTATT |
| IFN-β |  | ACGCCGCATTGACCATCTAT | GTCTCATTCCAGCCAGTGCTA |
| GAPDH |  | GAAGGTGAAGGTCGGAGTC | GAAGATGGTGATGGGATTTC |


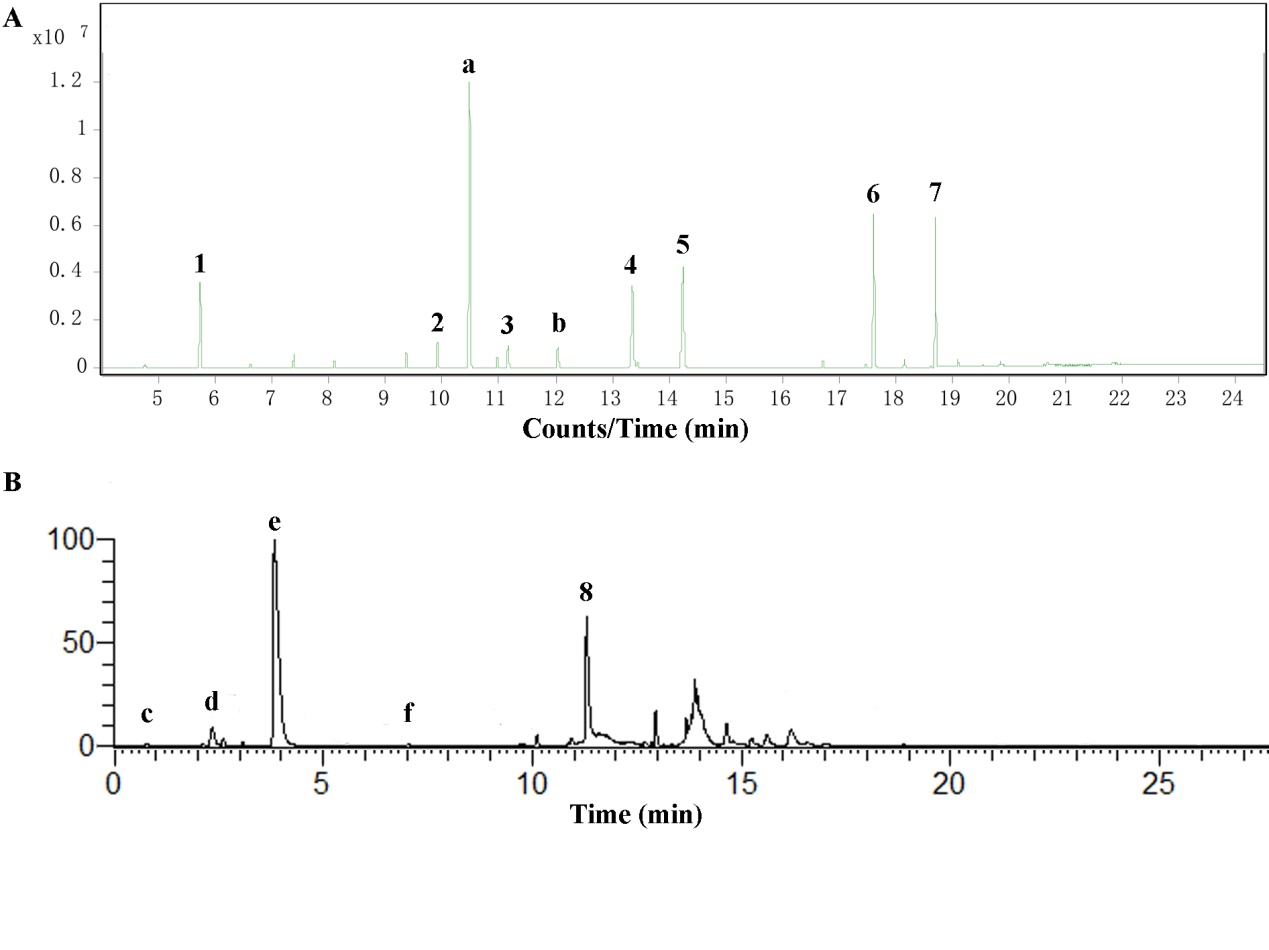


Figure S1 GC-MS and LC-MS chromatogram of reference substances

A: The GC-MS chromatogram of (1) eucalyptol, (2) camphor, (3) menthol, (4) methyl salicylate, (5) trans-anethole, (6) cinnamaldehyde, (7) eugenol, (a) dimethyl sulfoxide, (b) borneol. B: The LC-MS chromatogram of (8) ligustilide, (c) kinsenoside, (d) hesperidin, (e) nitidine chloride, (f) ginsenoside Rb1.

A

B
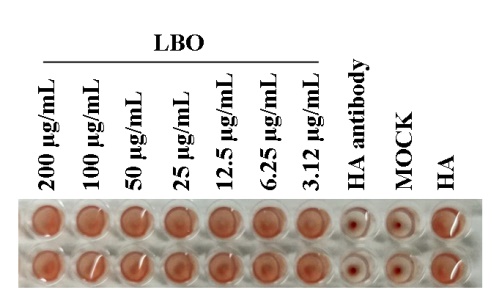


C**
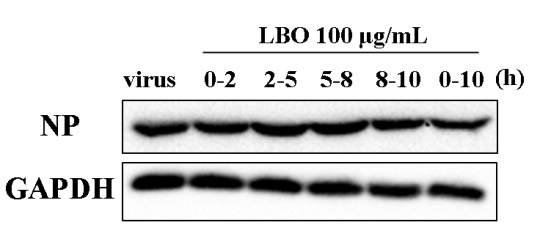
**

Figure S2 The influences of LBO on the influenza virus

The influenza virus A/WSN/1933 (H1N1) and fluorescent methyl umbelliferone N- acetyl neuraminic acid based assay was used to test the neuraminidase inhibition of LBO, zanamivir was used as a positive control. Chicken red blood cells and hemagglutinin (HA) was used to test the hemagglutination inhibition of LBO, HA antibody was used as a positive control. The NP protein was used to test the influence of LBO on the replication of IVA. After being infected by influenza virus A/WSN/1933 (H1N1), the MDCK cells were treated with 100 μg/mL LBO at different time intervals of one cycle of influenza virus replication (0-2, 2-5, 5-8, 8-10 and 0-10 h), the samples were collected and tested at 10 h post infection. A: The influences of LBO on the neuraminidase activity. B: The influences of LBO on the hemagglutination activity. C: The influence of LBO on the replication of IVA during the whole replication cycle.
